# Invasive Species as Hosts of Zoonotic Infections: The Case of American Mink (*Neovison vison*) and *Leishmania infantum*

**DOI:** 10.3390/microorganisms9071531

**Published:** 2021-07-18

**Authors:** Iris Azami-Conesa, Jose Sansano-Maestre, Rafael Alberto Martínez-Díaz, María Teresa Gómez-Muñoz

**Affiliations:** 1 Department of Animal Health, Faculty of Veterinary Sciences, Complutense University of Madrid, 28040 Madrid, Spain; irisazami@ucm.es; 2 Department of Animal Health and Public Health, Catholic University of Valencia, 46002 Valencia, Spain; jose.sansano@ucv.es; 3 Department of Preventive Medicine and Public Health, and Microbiology, Faculty of Medicine, Autonomous University of Madrid, 28029 Madrid, Spain

**Keywords:** American mink, ITS, kDNA, leishmaniasis, one health, hosts, Repeat region, SSUrRNA, wild carnivore, zoonosis

## Abstract

*Leishmania infantum* produces an endemic disease in the Mediterranean Basin that affects humans and domestic and wild mammals, which can act as reservoir or minor host. In this study, we analyzed the presence of the parasite in wild American minks, an invasive species in Spain. We screened for *L. infantum* DNA by PCR using five primer pairs: Two targeting kinetoplast DNA (kDNA), and the rest targeting the ITS1 region, the small subunit of ribosomal RNA (SSU) and a repetitive sequence (Repeat region). The detection limit was determined for each method using a strain of *L. infantum* and a bone marrow sample from an infected dog. PCR approaches employing the Repeat region and kDNA (RV1/RV2 primers) showed higher sensitivity than the other PCR methods when control samples were employed. However, only PCR of the Repeat region and nested PCR of SSU (LnSSU) detected the parasite in the samples, while the other three were unable to do so. The majority of the analyzed animals (90.1%) tested positive. American mink may act as an incidental host of the disease for other mammals and should be further investigated, not only for their negative impact on the local fauna, but also as carriers of zoonotic diseases.

## 1. Introduction

The American mink (*Neovison vison*) is a mustelid native of North America that was introduced in Europe for fur farming. Throughout recent decades, accidental or deliberate escape of mink from farms caused the establishment of stable feral populations. In Spain, the first observation of feral animals was detected at the end of the 1970s, and at present, there are stable populations distributed throughout the northwest and east of the Iberian Peninsula (North of Galicia, Cantabria, and Teruel-Castellón) [1]. The presence of this mammal on the banks of rivers supposes a strong competition for resources with other aquatic carnivores such as the European otter (*Lutra lutra*) or the endangered European mink (*Mustela lutreola*) [2]. For this reason, American mink is included in the European list of invasive species and is subjected to control and eradication programs [3]. Moreover, American mink can act as a carrier of multiple pathogens, including several nematode species [4], protozoans such as *Toxoplasma gondii* [5], and viruses such as influenza A [6] and SARS-CoV-2 [7]. These pose potential health risks for man and also for autochthonous freshwater carnivores in areas where there is an overlap in the distribution with this alien species.

*Leishmania infantum* is one of the etiological agents of zoonotic leishmaniasis, which is an endemic parasite of the Mediterranean Basin. It is mainly transmitted by phlebotomine sandfly bites [8], although other ways of transmission, such as sexually or transplacentally, have been proven in dogs, which are considered the main reservoir for human *Leishmania* parasites [9]. The disease has several forms, with the cutaneous (CL) and visceral (VL) forms being the most common. More than one billion people are at risk of leishmaniasis in endemic areas around the world, and 30,000 new cases of VL are estimated every year. In addition to the high number of infected humans and animals that develop the disease, there are also a significant amount of people, dogs, and other animals carrying asymptomatic infections [10].

Occasionally, conditions that favor the expansion of the disease, including spatial and temporal coincidence of humans, vectors, and potential reservoirs appear, lead to human outbreaks. The ideal habitats for vector expansions are warm areas rich in organic material, such as caves or burrows where animals breed, including wild mammals [11].

When leishmaniasis outbreaks appear, an exhaustive search of potential reservoirs and vectors begins, and monitoring activities can reveal new hosts for the parasite. In the case of Spain, an outbreak starting in 2009 in Madrid highlighted the importance of wildlife as potential reservoirs of the disease, specifically lagomorphs [12]. Thenceforward, several studies reported the presence of *Leishmania* in different species of wildlife, including wolfs, foxes, mustelids, bats, hedgehogs, and rodents [13,14,15,16,17,18]. Despite the number of articles studying the role of wildlife as reservoirs of leishmaniasis, invasive species are not widely studied thus far. Invasive species are relevant not only for other wild species, but also for humans, considering the growing approach to urban places, and the potential role as reservoirs of vector-borne pathogens [19]. The American mink is in the order Carnivora, which includes many species considered as reservoirs and/or hosts suffering the disease. The presence of *L. infantum* has been reported in mink farms in Greece [20,21] and in one wild American mink killed by a car in Spain [17], which suggest its role as a host of *L. infantum*.

In this article, we explored the role of wild communities of American mink as a host of *L. infantum* by PCR using five primer pairs to detect the DNA of the parasite.

## 2. Materials and Methods

### 2.1. Samples and Area of Study

**Figure 1:**
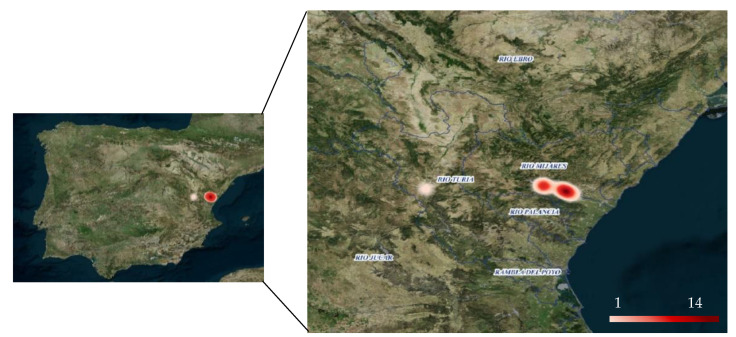
Geographic area where the animals were captured. The color intensity indicates the number of animals captured in each area (see bar on bottom).

Spleen samples from American minks of three different river basins (Mijares, Palancia, and Turia) located in the Valencian Community (Spain) were analyzed (Figure 1). The animals were hunted in the context of a European LIFE project for the conservation of the European mink (LIFE Lutreola Spain, LIFE13 NAT/ES/001171), which include the eradication of the American mink among its objectives, due to the high menace they impose on the autochthonous fauna. The American mink is categorized as an alien invasive species in Europe [22], and European [23], National [24], and regional legislation [25] regulate the control and/or eradication programs of invasive species, such as *Neovison vison*, according to the status in each country. One of the objectives of the LIFE13 NAT/ES/001171 and the Spanish strategy for control and eradication of the American mink is to capture and euthanize as many animals as possible. The total number of animals captured in the Valencian community in previous control programs was 220 in 8–10 years, which means that the present sample approaches the highest number of animals possibly subject to eradication per year, although a precise number of animals in the area is not available. More information on the distribution of stable populations of feral American minks in Spain can be found elsewhere [26]. The animals were captured from February to October 2016 and were humanely euthanized, minimizing pain and distress, according to veterinary or control program criteria (animals which could not be reintroduced into the wild) and following the recommendations of the Spanish strategy for the control and eradication of the American mink. Euthanasia was carried out by the veterinary staff of the wildlife recovery center “La Granja de El Saler” (Valencia, Spain), following the AVMA guidelines for euthanasia of animals [27]. Biometric measurements were taken from each animal and a necropsy was performed. A sample from the spleen of each animal was kept frozen (−80 °C) until further analysis. 

### 2.2. Culture of Leishmania and Determination of the Detection Limit

A culture of the strain “JPC” (MCAN/ES/98/LLM-722) of *L. infantum* generously supplied by Dr. J.M. Requena was used to calculate the detection limit of the PCR protocols employed. The isolate was grown in RPMI containing 15% fetal bovine serum (Sigma, St. Louis, MO, USA) and 10 µg/mL of hemin (Acros Organics, Geel, Belgium). In the exponential phase of the culture (day 3 post-inoculation), promastigotes were counted in a Neubauer chamber using 1:1 Trypan blue (4%) in 10% formaldehyde. One million promastigotes were employed for DNA isolation. The parasites were recovered by centrifugation at 800× *g* for 5 min. The sediment was washed three times with phosphate-buffered saline (PBS) solution, followed by centrifugation in the same manner. We isolated DNA from the pellets obtained using a commercial DNA extraction kit (NZY Tissue gDNA Isolation kit; NZY Tech, Lisbon, Portugal), following the manufacturer’s protocol. Bound DNA was eluted in 50 µL of elution buffer and stored at −20 °C until use. 

The limit of detection was evaluated by conventional PCR, using four different targets (Repeat region, kDNA, ITS1, and SSUrRNA) by 1/10 serial dilutions, up to 10^−7^-fold dilutions, using DNA from the *L. infantum* culture. The initial DNA concentration was quantified by spectrophotometry (260/280 nm ratio) using a basic Eppendorf BioSpectrometer^®^ (Eppendorf AG, Hamburg, Germany), and 20 µg/mL was obtained.

To evaluate the detection limit of each PCR protocol, and also as a positive control of each PCR reaction, DNA from the bone marrow of a dog infected with *L. infantum,* kindly supplied by Prof. Luis Cardoso (UTAD, Vila Real, Portugal), was used. Again, 1/10 serial dilutions, up to 10^−7^-fold dilutions, were employed to compared the five PCR protocols. 

### 2.3. DNA Isolation of Spleen Samples and PCR

DNA was isolated from 15 mg of spleen tissue, employing the NZY Tissue gDNA Isolation kit, according to the tissue protocol instructions, and later eluted in 50 µL of elution buffer. Positive and negative controls (*L. infantum* culture and water, respectively) were included in each batch.

Each DNA sample was subjected to five protocols of PCR to detect the parasite: Two protocols targeting kinetoplast DNA (kDNA) [28,29], one protocol targeting the ITS region [30], a nested PCR targeting the small subunit of the ribosomal RNA (LnPCR SSU) [31,32], and a protocol amplifying a Repeat region of the parasite [33]. In all cases, the reaction was carried out in 25 µL, employing 12.5 µL of the HotStart Supreme NZY Taq II (NZY Tech, Lisbon, Portugal) and 1 µL of each primer at 10 µM. For all of the protocols, 2.5 µL of DNA was employed, except for the SSU, which used 5 µL of DNA in the first PCR and 5 µL of 1/40 dilution of PCR product in the second PCR. The specific information of each target, including the temperature profiles following adaptation suggested by manufacturer, and primers sequences is available in Table 1. An initial step of 95 °C for 5 min was used to activate the enzyme and a final step of 72 °C for 10 min for elongation was used in all of the protocols. As a positive control for the amplifications, DNA from the abovementioned bone marrow was employed, whilst water was used as a negative control. Electrophoresis was carried out in 1.5% agarose gel stained with GelRed^®^ Nucleic Acid Gel Stain (Biotium Inc., San Francisco, CA, USA) and later visualized under UV light. After each PCR protocol, the samples were considered positive if a band of the expected size was observed in the gel. 

### 2.4. Sequencing

Positive samples were cleaned using an ExoSAP-IT commercial kit (Exonuclease I/Shrimp Alkaline Phosphatase, Applied Biosystems, Foster City, CA, USA). Amplicons were sequenced in both directions at the Genomic Unit of Complutense University of Madrid with a 3730 × L DNA analyzer (Applied Biosystems), using a BigDye Terminator Cycle Sequencing kit v3.1 (Applied Biosystems). The obtained sequences were aligned employing Lasergen SeqMan software version 7.0.0 (DNASTAR, Madison, WI, USA) and manually checked. The consensus sequences were compared with available sequences using the BLAST algorithm of NCBI (https://blast.ncbi.nlm.nih.gov/Blast.cgi (accessed on 16 April 2021). Sequences of more than 200 bp were submitted to GenBank for accession number identification.

## 3. Results

### 3.1. Samples

In total, samples from 22 captured animals were obtained during the eradication program of invasive species in 2016, in the context of a LIFE project to protect the European mink. Data were recorded from each animal, including date of capture, age, sex, weight, length, and pregnancy in the case of females (Table 2). With the exception of one juvenile male, the rest of the individuals were adults—nine males and 10 females, two of them pregnant. The animals did not show any clinical signs of disease.

### 3.2. PCR Detection Limit

The amount of DNA amplified with each protocol varied among the tested procedures. The most sensitive protocols were PCR of the Repeat region and PCR of kDNA following the protocol of Lachaud et al. [28], while the other three protocols varied between the culture and the bone marrow samples from a positive dog.

#### 3.2.1. PCR Detection Limit Using a Reference Strain

PCRs of the Repeat region and kDNA (RV1/RV2 primers) were able to detect 0.5 promastigotes of *L. infantum* (equivalent to 0.05 pg DNA) (Table 3), while LnPCR of the SSU amplified a minimum of one promastigotes of the parasite (0.1 pg DNA), since a doubled amount of DNA was employed in LnPCR SSU. PCRs of the ITS region and kDNA (13A/13B primers) were able to detect a minimum of five promastigotes (0.5 pg DNA). 

#### 3.2.2. PCR Detection Limit Using DNA from the Bone Marrow of an Infected Dog

When DNA from the bone marrow of a positive dog was employed, PCR of the Repeat region and of kDNA (RV1/RV2 primers) were again the most sensitive protocols, displaying positive results even at a 10^−4^-fold dilution from the original DNA (Table 4). PCR of the ITS region showed positive results up to 10^−3^ dilution from the original DNA, while PCR of kDNA (13A/13B primers) and LnPCR SSU appeared less sensitive, amplifying only at a maximum of a 10^−2^-fold dilution from the original DNA sample. 

### 3.3. PCR Results from Mink Spleen Samples

In total, 22 samples were analyzed by the five PCR protocols. Only PCR of the Repeat region and LnPCR of SSU rendered positive results, while the other three techniques amplified only the positive controls included in each reaction set. Nineteen samples were positive to PCR of the Repeat region, while only two samples were positive to LnPCR SSU (Table 5).

### 3.4. Sequences Obtained

The sequences obtained in this study were deposited to GenBank under accession numbers MW972061–MW972074, MW945401, and MW945402. Fourteen 217 bp sequences from the Repeat region were obtained, while four samples displayed shorter sequences and could not be sent to GenBank. The obtained sequences from the Repeat region shared 100% identity with *L. infantum* sequence L42479. The two 322 bp sequences from the SSU region shared 100% identity with *L. infantum* sequence MN757921 from an isolate obtained from a dog.

## 4. Discussion

Herein, we reported a high percentage of animals infected with the *L. infantum*, but according to the results obtained employing the different PCR targets, and considering the detection limit obtained, it is probable that the animals carried a low amount of parasites in their bodies. All positive animals, except one, were adults, and they had exposure to at least one full season of sandfly activity. An explanation for the low parasite load could be that the context in which minks interact with sandfly populations in the wild limits the amount of bites they are exposed to and, consequently, the dose of promastigotes that each animal received. Alternatively, a transient infection with the parasite could be the cause of the low parasitic load. American minks have a lifespan of 10–12 years in the wild and they live in colonies, which facilitates host–sandfly contact. These characteristics, together with the high percentage of infection, the presence of *Phlebotomus perniciosus* in the area [34], and the identity of *L. infantum* DNA sequence from a dog, support their role as a host of the parasite and they should be considered for future studies.

There are a large number of domestic and wild animals in Europe, besides dogs, in which *L. infantum* has been described [35]. Among them, wild carnivores are the most studied and are considered suitable reservoirs for the parasite, since specific DNA has been detected in many of them, including canids and felids [35]. In addition, several species of mustelids have been reported with *L. infantum* DNA: European mink (*Mustela lutreola*), Eurasian otter (*Lutra lutra*), Beech marten (*Martes foina*), European pine marten (*Martes martes*), domesticated ferrets (*Mustela putorius furo*), European badger (*Meles meles*), and European polecat (*Mustela putorius*) [13,15,21,36,37,38].

A high rate of infection has been reported in farmed American minks (21.4% of analyzed animals), even at a young age [20]. In another study, 20% of older farmed minks were positive by serology [21], although lower values were found employing PCR of the ITS region (2.1%). Stress and conditions of the farms (temperature and humidity) may favor the development and expansion of sandflies. Whereas the presence of *L. infantum* seems to be frequent in farmed animals, only one study detected a wild American mink infected with the parasite by qPCR in Spain, but the authors only tested one animal [17]. In all cases, parasites were detected in samples from the spleen, and from the liver of one of them. Apart from this single report of DNA in one exemplar, the role of American minks as incidental host of the parasite in wild communities has not been investigated until the present article.

In this study, we employed different targets to detect the parasite as a multi-locus approach to assess the identity of the obtained sequences in comparison to other *L. infantum* sequences from humans and animals. Unfortunately, only two of them displayed positive results. Under our conditions, PCR using the Repeat region and kDNA (RV1 and RV2 primers) were the most sensitive techniques for detecting DNA from parasite cultures and from the bone marrow of a positive dog. Using DNA samples from cultures, in which the amount of parasites is easier to quantify, LnPCR of SSU showed the same limit of sensitivity. When employing PCR from the ITS region, a lower percentage of positivity has also been reported by other authors, including lower values of PCR detection in farmed American minks compared to serology [21]. Lower sensitivity employing the ITS target was also found in samples from hedgehogs, while PCR using kDNA and the Repeat region as the target displayed better results, with 6/24 samples positives by PCR kDNA and 13/26 positives by nested PCR of the Repeat region [39]. In agreement with the results obtained by Chemkhi et al. [39], PCR amplifying the Repeat region was the best approach to detect positivity in the samples employed in the present study.

Other authors have also compared different protocols of amplification, with the results varying between studies, although most of them agreed in pointing out that kDNA is a high-sensitivity target due to its multicopy nature, with thousands of maxicircles and several dozen of minicircles [40]. Lachaud et al. [28] compared six different primer pairs to detect the parasite in the blood of infected animals, three of them targeting nuclear DNA, and three more targeting mitochondrial DNA (kinetoplast minicircles). The authors found a limit of detection of 0.2 parasites per reaction tube employing the primers targeting the Repeat region, the same value reported in the present study using the same primer pairs. However, primers 13A and 13B were more sensitive, according to the authors, while in the present study, they were the less sensitive. A strong inhibition was reported by Lachaud et al. [28] when employing PCR of the Repeat region, but in the present study, no signs of inhibition were found, a fact that could be due to differences between the polymerases or DNA extraction protocols applied in each study. When tissues are employed to isolate DNA, results may vary from one individual to another, or even between animal species [28]. Indeed, spleen samples are full of inhibitors or competitors, and manufacturers recommend in many protocols to reduce the amount of tissue processed.

Albuquerque et al. [41] compared four PCR methods, two targeting kinetoplast DNA and two targeting nuclear DNA. According to the authors, LnPCR SSU was able to detect a higher number of samples from the bone marrow of clinically and sub-clinically infected animals, while ITS1 showed the lowest level of detection. The two mentioned PCR protocols were also employed in the present study, and the obtained results agree with those reported by the authors. None of the primer pairs targeting kDNA were employed in the present study and no comparison between results could be done.

Concerning the negative results obtained with both kDNA PCR protocols, they might be due to negligible changes in DNA sequences being enough to avoid amplification from the samples, since they represent a more variable target than the Repeat region or the SSU region [40]. Indeed, several approaches using kDNA as targets can be found in the literature and the results varied with the primers employed. De Oliveira et al. [42] reported that primers 13A and 13B had the worst performance among four primers derived from kDNA sequences, and employing DNA from parasite strains, a fact that is in agreement with the results obtained in the present study.

Taking into account our results, we believe that the employment of more than one target to detect *L. infantum* is necessary. Different sensitivities of the protocols, or minimal differences in the DNA sequences of more variable targets, could interfere with the results.

This is the first report of the detection of *L. infantum* in communities of wild American minks. The American mink is an invasive species that menaces the European mink, but it also harbors several zoonotic infections [4,5,6,7,17,20,21]. For these reasons, they could be used as sentinel species to monitor the epidemiological status of zoonotic pathogens in the regions where they inhabit, and this approach could be included in European mink conservation programs.

## Figures and Tables

**Table 1:** PCR targets and primer pairs and conditions employed in each PCR protocol.

Target	Oligonucleotides (5′-3′)	Expected Size of the Amplicon	PCR Conditions	N° Cycles	Reference
Repeat region	T2 (CGGCTTCGCACCATGCGGTG) B4 (ACATCCCTGCCCACATACGC)	250 bp	94 °C—30″ 61 °C—30″ 72 °C—15″	35	[33]
kDNA	RV1 (CTTTTCTGGTCCCGCGGGTAGG) RV2 (CCACCTGGCCTATTTTACACCA)	140 bp	94 °C—30″ 62 °C—30″ 72 °C—20″	35	[28]
kDNA	13A (GTGGGGGAGGGGCGTTCT) 13B (ATTTTACACCAACCCCCAGTT)	120 bp	94 °C—30″ 60 °C—30″ 72 °C—20″	30	[29]
ITS1	LITSR (CTGGATCATTTTCCGATG) L5.8S (TGATACCACTTATCGCACTT)	320 bp	94 °C—30″ 53 °C—30″ 72 °C—15″	35	[30]
SSU	Ext R221 (GGTTCCTTTCCTGATTTACG) Ext R332 (GGCCGGTAAAGGCCGAATAG)	603 bp	94 °C—30″ 60 °C—30″ 72 °C—30″	35	[31]
	Int R223 (TCCATCGCAACCTCGGTT) Int R333 (AAAGCGGGCGCGGTGCTG)	358 bp	94 °C—30″ 65 °C—30″ 72 °C—30″	32	[32]

Ext, external primers; Int, internal primers.

**Table 2:** Data from the animals captured.

Animal ID	Date	Sex	Age	Weight (g)	Length (cm)	River
68/16	8 March 2016	F	Ad	680	57	Mijares
26/16	10 February 2016	F	Ad	689	58	Mijares
192/16	n.d.	n.d.	n.d.	n.d.	n.d.	n.d.
56/16	1 March 2016	M	Ad	1180	62	Mijares
23/16	10 February 2016	F	Ad	793	58.9	Mijares
197/16	20 October 2016	M	Ad	950	63	Palancia
66/16	6 March 2016	F	Ad	600	51	Mijares
201/16	22 October 2016	F	Ad	660	55.5	Palancia
55/16	1 March 2016	M	Ad	980	56	Mijares
41/16	18 February 2016	M	Ad	901	59	Mijares
62/16	4 March 2016	F	Ad	640	48	Mijares
1023/16	22 May 2016	M	Juv	878	62	Turia
77/16	14 March 2016	M	Ad	1180	64	Mijares
212/16	26 October 2016	F	Ad	650	58	Palancia
213/16	27 October 2016	M	Ad	810	58	Palancia
35/16	16 February 2016	M	Ad	1109	63.5	Mijares
29/16	14 February 2016	F *	Ad	721	57	Mijares
37/16	17 February 2016	M	Ad	1604	69	Mijares
30/16	14 February 2016	F *	Ad	583	52	Mijares
28/16	14 February 2016	F	Ad	637	56	Mijares
331/16	n.d.	n.d.	n.d.	n.d.	n.d.	n.d.
1001/16	n.d.	n.d.	n.d.	n.d.	n.d.	n.d.

n.d., no data recorded; F, female; Juv, juvenile; M, male; * pregnant.

**Table 3:** PCR amplification from the DNA obtained from the *L. infantum* strain using the protocols employed for the detection and identification of *L. infantum*.

		Ten-Fold Dilutions from the Original DNA Sample						
PCR Protocol	DNA from Culture (20 µg/mL)	10^−1^	10^−2^	10^−3^	10^−4^	10^−5^	10^−6^	10^−7^
PCR Repeat region	+	+	+	+	+	+	−	−
PCR kDNA(RV1/RV2)	+	+	+	+	+	+	−	−
LnPCR SSU	+	+	+	+	+	+	−	−
PCR kDNA(13A/13B)	+	+	+	+	+	−	−	−
PCR ITS1	+	+	+	+	+	−	−	−

PCR of the kDNA region includes primers in parentheses to differentiate the two protocols applied. The first column includes the results with DNA without dilution, and each column adds 1/10 dilution from the original DNA. +, positive; −, negative.

**Table 4:** PCR amplification from bone marrow DNA of a positive dog using the five PCR protocols employed for detection and identification of *L. infantum*.

		Ten-Fold Dilutions from the Original DNA Sample						
PCR Protocol	Bone Marrow DNA	10^−1^	10^−2^	10^−3^	10^−4^	10^−5^	10^−6^	10^−7^
PCR Repeat region	+	+	+	+	+	−	−	−
PCR kDNA(RV1/RV2)	+	+	+	+	+	−	−	−
LnPCR SSU	+	+	+	−	−	−	−	−
PCR kDNA(13A/13B)	+	+	+	−	−	−	−	−
PCR ITS1	+	+	+	+	−	−	−	−

PCR of kDNA region includes primers in parentheses to differentiate the two protocols applied. The first column includes results with DNA without dilution, and each column adds 1/10 dilution from the original DNA. +, positive; −, negative.

**Table 5:** PCR amplification from spleen samples using the five PCR protocols used for the detection and identification of *L. infantum*. PCR of the kDNA region includes primers in parentheses to differentiate the two protocols employed.

Animal ID	PCR Repeat Region	LnPCR SSU	PCR kDNA (RV1/RV2)	PCR kDNA (13A/13B)	PCR ITS
68/16	+	−	−	−	−
26/16	+	−	−	−	−
192/16	+	−	−	−	−
56/16	+ *	−	−	−	−
23/16	+	−	−	−	−
197/16	+	−	−	−	−
66/16	+	−	−	−	−
201/16	+	−	−	−	−
55/16	+	−	−	−	−
41/16	+	−	−	−	−
62/16	+	−	−	−	−
1023/16	+	−	−	−	−
77/16	+ *	−	−	−	−
212/16	−	−	−	−	−
213/16	+ *	−	−	−	−
35/16	−	−	−	−	−
29/16	+	+	−	−	−
37/16	+	−	−	−	−
30/16	+ *	+	−	−	−
28/16	+	−	−	−	−
331/16	+	−	−	−	−
1001/16	+ *	−	−	−	−

* Weak PCR band and short sequence; +, positive; −, negative.

## Data Availability

Sequence data are available from GenBank (https://www.ncbi.nlm.nih.gov/genbank/) under accession numbers MW972061–MW972074, MW945401 and MW945402.
